# Supplementary material for: Universally valid reduction of multiscale stochastic biochemical systems using simple non-elementary propensities
Source: PLoS Comput Biol. 2021 Oct 18;17(10):e1008952. doi: 10.1371/journal.pcbi.1008952 (PMC8562860; doi:10.1371/journal.pcbi.1008952)
Supplement: S1 Appendix — (PDF) [file pcbi.1008952.s001.pdf]

S1 Appendix for Universally valid reduction of multiscale stochastic biochemical systems using simple non-elementary propensities

Yun Min Song[1,2◑], Hyukpyo Hong[1,2◑], Jae Kyoung Kim[1,2*]

1 Department of Mathematical Sciences, Korea Advanced Institute of Science and Technology, Daejeon 34141, Republic of Korea
2 Biomedical Mathematics Group, Institute for Basic Science, Daejeon 34126, Republic of Korea

◑These authors contributed equally to this work.
* jaekkim@kaist.ac.kr

Supplementary Methods

Validity of the stochastic model reduction using the stochastic QSSA

Here, we show that an accurate approximation for a stochastic model containing a rapid reversible binding (Eq. (1)) can be obtained by replacing the fast variables (A, B, and C) representing the numbers of A, B, and C in Eq. (1) with their stochastic QSSAs ($\langle A\rangle$, $\langle B\rangle$, and $\langle C\rangle$) [1–4].

Let $P(n;t)$ represent the probability of being in state n at time t. If a system contains some reactions that are faster than the others, n can be partitioned into n_f and n_s denoting the states of fast species whose copy numbers are changed by the fast reactions and slow species whose copy numbers are not changed by the fast reactions, respectively. Then the chemical master equation (CME) for this system is

$$\begin{aligned}\frac{dP(n_f,n_s;t)}{dt} =&\sum_{j=1}^{N_f} a_j^f(n_f-v_{f,j}^f,n_s)P(n_f-v_{f,j}^f,n_s;t)\\&-a_j^f(n_f,n_s)P(n_f,n_s;t)\\&+\sum_{i=1}^{N_s} a_i^s(n_f-v_{f,i}^s,n_s-v_{s,i}^s)P(n_f-v_{f,i}^s,n_s-v_{s,i}^s;t)\\&-a_i^s(n_f,n_s)P(n_f,n_s;t),\end{aligned} \tag{S1}$$

where N_f and N_s are the numbers of fast and slow reactions, respectively, and a_j^f and a_i^s are the propensity functions of the jth fast and the ith slow reactions, respectively. Furthermore, $v_{f,j}^f$, $v_{f,i}^s$, and $v_{s,i}^s$ are the stoichiometric vectors associated with the fast species of the jth fast reaction, the fast species of the ith slow reaction, and the slow species of the ith slow reaction, respectively. Note that there is no $v_{s,j}^f$ in Eq. (S1) because the number of slow species is not affected by the fast reactions.

When a multiscale stochastic model contains the rapid reversible binding between A and B forming complex C and all the other reactions are slower than the reversible binding, n_f consists of the variables A, B, and C, the numbers of A, B, and C, respectively, and n_s consists of the variables that correspond to the other species.

Note that the rapid reversible binding reactions does not change the total numbers (i.e., both the free form and the complex form) of A species and B species ($A_{\mathrm{T}} = A + C$ and $B_{\mathrm{T}} = B + C$).. Thus, by changing the variables, $A = A_{\mathrm{T}} - C$, $B = B_{\mathrm{T}} - C$, we can eliminate A and B in n_f while adding A_{T} and B_{T} in n_s. Then, because n_f contains only C, the CME (Eq. (S1)) becomes

$$\begin{aligned}\frac{dP(C, n_s; t)}{dt} =& \frac{k_{\mathrm{f}}}{\Omega}(A_{\mathrm{T}} - C + 1)(B_{\mathrm{T}} - C + 1)P(C - 1, n_s; t) \\ &- \frac{k_{\mathrm{f}}}{\Omega}(A_{\mathrm{T}} - C)(B_{\mathrm{T}} - C)P(C, n_s; t) \\ &+ k_{\mathrm{b}}(C + 1)P(C + 1, n_s; t) \\ &- k_{\mathrm{b}}C \cdot P(C, n_s; t) \\ &+ \sum_{i=1}^{N_s} a_i^s(C - v_{f,i}^s, n_s - v_{s,i}^s)P(C - v_{f,i}^s, n_s - v_{s,i}^s; t) \\ &\quad - a_i^s(C, n_s)P(C, n_s; t),\end{aligned} \tag{S2}$$

where Ω is the system volume.

Since $P(C, n_s; t) = P(C|n_s; t)P(n_s; t)$, where $P(C|n_s; t)$ represents the probability of C at time t conditioned on n_s, Eq. (S2) can be represented as

$$\begin{aligned}&\frac{dP(C|n_s; t)}{dt}P(n_s; t) + P(C|n_s; t)\frac{dP(n_s; t)}{dt} \\ &= \frac{k_{\mathrm{f}}}{\Omega}(A_{\mathrm{T}} - C + 1)(B_{\mathrm{T}} - C + 1)P(C - 1|n_s; t)P(n_s; t) \\ &- \frac{k_{\mathrm{f}}}{\Omega}(A_{\mathrm{T}} - C)(B_{\mathrm{T}} - C)P(C|n_s; t)P(n_s; t) \\ &+ k_{\mathrm{b}}(C + 1)P(C + 1|n_s)P(n_s; t) \\ &- k_{\mathrm{b}}C \cdot P(C|n_s; t)P(n_s; t) \\ &+ \sum_{i=1}^{N_s}[a_i^s(C - v_{f,i}^s, n_s - v_{s,i}^s)P(C - v_{f,i}^s|n_s - v_{s,i}^s; t)P(n_s - v_{s,i}^s; t) \\ &\quad - a_i^s(C, n_s)P(C|n_s; t)P(n_s; t)].\end{aligned} \tag{S3}$$

In a short timescale, we can assume that only the fast binding and unbinding reactions occur, so the slow variables do not change (i.e., $\frac{dP(n_s)}{dt} = 0$). Then we can rewrite Eq. (S3) as

$$\begin{aligned}\frac{dP(C|n_s; t)}{dt} =& \frac{k_{\mathrm{f}}}{\Omega}(A_{\mathrm{T}} - C + 1)(B_{\mathrm{T}} - C + 1)P(C - 1|n_s; t) \\ &- \frac{k_{\mathrm{f}}}{\Omega}(A_{\mathrm{T}} - C)(B_{\mathrm{T}} - C)P(C|n_s; t) \\ &+ k_{\mathrm{b}}(C + 1)P(C + 1|n_s; t) \\ &- k_{\mathrm{b}}C \cdot P(C|n_s; t).\end{aligned} \tag{S4}$$

Then, while n_s is fixed, C evolves following Eq. (S4) and thus equilibriate to its conditional stationary distribution $P(C|n_s)$, which satisfies

$$\begin{aligned}0 =& \frac{k_{\mathrm{f}}}{\Omega}(A_{\mathrm{T}} - C + 1)(B_{\mathrm{T}} - C + 1)P(C - 1|n_s) \\ &- \frac{k_{\mathrm{f}}}{\Omega}(A_{\mathrm{T}} - C)(B_{\mathrm{T}} - C)P(C|n_s) \\ &+ k_{\mathrm{b}}(C + 1)P(C + 1|n_s) \\ &- k_{\mathrm{b}}C \cdot P(C|n_s).\end{aligned} \tag{S5}$$

In a long timescale, C has already reached the conditional stationary distribution $P(C|n_s)$ (i.e., $P(C|n_s;t) \approx P(C|n_s)$ and thus $\frac{dP(C|n_s;t)}{dt} = 0$). Then by summing both sides of Eq. (S3) over all possible states of C, we get the following:

$$\begin{aligned}
&\frac{dP(n_s;t)}{dt}\sum_C P(C|n_s) \\
&\qquad =\sum_C [\frac{k_\mathrm{f}}{\Omega}(A_\mathrm{T}-C+1)(B_\mathrm{T}-C+1)P(C-1|n_s)P(n_s;t) \\
&\qquad\quad -\frac{k_\mathrm{f}}{\Omega}(A_\mathrm{T}-C)(B_\mathrm{T}-C)P(C|n_s)P(n_s;t) \\
&\qquad\quad + k_\mathrm{b}(C+1)P(C+1|n_s)P(n_s;t) \\
&\qquad\quad - k_\mathrm{b}C\cdot P(C|n_s)P(n_s;t)] \\
&\qquad +\sum_{i=1}^{N_s}\sum_C [a_i^s(C-v_{f,i}^s, n_s-v_{s,i}^s)P(C-v_{f,i}^s|n_s-v_{s,i}^s)P(n_s-v_{s,i}^s;t) \\
&\qquad\quad - a_i^s(C,n_s)P(C|n_s)P(n_s;t)].
\end{aligned} \tag{S6}$$

In Eq. (S6), the summation of $P(C|n_s)$ in the LHS is one as it is a probability distribution, and the first summation term (i.e., four lines) in the RHS becomes zero by Eq. (S5). Then we get the following reduced CME that consists of only the slow variables (n_s) and the slow reactions:

$$\frac{dP(n_s)}{dt} = \sum_{i=1}^{N_s} \overline{a}_i^s(n_s - v_{s,i}^s)P(n_s - v_{s,i}^s) - \overline{a}_i^s(n_s)P(n_s), \tag{S7}$$

where $\overline{a}_i^s(n_s) = \sum_C a_i^s(C,n_s)P(C|n_s)$, i.e., the stationary conditional expectation of the propensity functions for the slow reactions. When the slow reactions are uni- or bi-molecular reactions, each $\overline{a}_i^s$ is a linear function in terms of the fast variables. Then

$$\overline{a}_i^s(n_s) = \sum_C a_i^s(C,n_s)P(C|n_s) = a_i^s(\sum_C C\cdot P(C|n_s), n_s) = a_i^s(\langle C|n_s\rangle, n_s)$$

where $\langle C|n_s\rangle$ denotes the stationary average number of C conditioned on n_s. Then the reduced CME (Eq. (S7)) becomes

$$\frac{dP(n_s)}{dt} = \sum_{i=1}^{N_s} a_i^s(\langle C|n_s - v_{s,i}^s\rangle, n_s - v_{s,i}^s)P(n_s - v_{s,i}^s) - a_i^s(\langle C|n_s\rangle, n_s)P(n_s). \tag{S8}$$

Since the conditional stationary average number of C ($\langle C|n_s\rangle$) solely depends on A_T and B_T, $\langle C|n_s\rangle$ is the same as the stochastic QSSA, $\langle C\rangle$, defined in the main text. Thus, Eq. (S8), where the fast variables (A, B, C) are replaced with their stochastic QSSAs ($\langle A\rangle = A_\mathrm{T} - \langle C\rangle$, $\langle B\rangle = B_\mathrm{T} - \langle C\rangle$, $\langle C\rangle$), is a faithful approximation of a stochastic model containing a rapid reversible binding in a long timescale.

Exact bound for the Fano factor of A

In this section, we show that the Fano factor of A (F_{A}) is always less than 1. We first define $\langle A^n|a,b\rangle$ as the stationary n^{th} power moment of A conditioned on $A_{\mathrm{T}}=a$ and $B_{\mathrm{T}}=b$. Then for any positive integer a and b,

$$\begin{aligned} F_{\mathrm{A}} &= \frac{\langle A^2|a,b\rangle - \langle A|a,b\rangle^2}{\langle A|a,b\rangle} \\ &= \frac{\langle A^2|a,b\rangle}{\langle A|a,b\rangle} - \langle A|a,b\rangle. \end{aligned} \tag{S9}$$

When $a=1$, A can be only 0 and 1, so $\langle A^2|1,b\rangle = \langle A|1,b\rangle$, and thus $F_{\mathrm{A}} = 1 - \langle A|1,b\rangle < 1$. Now we assume that $a \geq 2$. From Theorem 2 in [5], we have

$$\langle A^{(r)}|a,b\rangle = K_{\mathrm{d}}^r \frac{F_0(a-r,b)}{F_0(a,b)} \tag{S10}$$

for any $a \geq r$ and $b \geq 0$ where $A^{(r)} = \frac{A!}{(A-r)!}$. Here, F_0 is a generating function defined by

$$F_0(b_1,b_2) = \sum_{\substack{k_1,k_2,k_3 \geq 0, \\ k_1+k_3=b_1, \\ k_2+k_3=b_2}} \frac{\lambda_1^{k_1}}{k_1!}\frac{\lambda_2^{k_2}}{k_2!}\frac{\lambda_3^{k_3}}{k_3!}$$

where $(\lambda_1,\lambda_2,\lambda_3)$ is any positive deterministic steady state of the reversible binding reaction [6]. Here, we set $(\lambda_1,\lambda_2,\lambda_3) = (K_{\mathrm{d}},1,1)$. Then for $r=1$,

$$\langle A|a,b\rangle = K_{\mathrm{d}} \frac{F_0(a-1,b)}{F_0(a,b)}, \tag{S11}$$

and for $r=2$,

$$\langle A^2|a,b\rangle - \langle A|a,b\rangle = K_{\mathrm{d}}^2 \frac{F_0(a-2,b)}{F_0(a,b)}. \tag{S12}$$

We can rewrite Eq. (S12) as

$$\begin{aligned} \langle A^2|a,b\rangle &= K_{\mathrm{d}}^2 \frac{F_0(a-2,b)}{F_0(a,b)} + \langle A|a,b\rangle \\ &= K_{\mathrm{d}} \frac{F_0(a-2,b)}{F_0(a-1,b)} \cdot K_{\mathrm{d}} \frac{F_0(a-1,b)}{F_0(a,b)} + \langle A|a,b\rangle \\ &= \langle A|a-1,b\rangle \cdot \langle A|a,b\rangle + \langle A|a,b\rangle. \quad (\because \text{Eq. (S11)}) \end{aligned}$$

Then by replacing $\langle A^2|a,b\rangle$ in Eq. (S9), we get

$$F_{\mathrm{A}} = 1 + \langle A|a-1,b\rangle - \langle A|a,b\rangle. \tag{S13}$$

Since $\langle A|a-1,b\rangle < \langle A|a,b\rangle$, we finally proved $F_{\mathrm{A}} < 1$.

While $\langle A|a-1,b\rangle < \langle A|a,b\rangle$ seems trivial, for the rigorousness, we provide the proof for this as follows. Since F_{A} is always positive, from Eq. (S13),

$$\langle A|a-1,b\rangle + 1 > \langle A|a,b\rangle, \tag{S14}$$

for all $a \geq 2$ and $b > 0$. From the recurrence relation derived in the previous work [5], we have

$$F_0(a,b) = \frac{K_{\mathrm{d}}+1+b-a}{a} F_0(a-1,b) + \frac{K_{\mathrm{d}}}{a} F_0(a-2,b),$$

which can be re-expressed as

$$\frac{F_0(a,b)}{F_0(a-1,b)} = \frac{K_\mathrm{d}+1+b-a}{a} + \frac{K_\mathrm{d}}{a}\frac{F_0(a-2,b)}{F_0(a-1,b)}.$$

By multiplying K_d to both sides, we have

$$\langle A|a,b\rangle^{-1} = K_\mathrm{d}\left(\frac{K_\mathrm{d}+1+b-a}{a} + \frac{1}{a}\langle A|a-1,b\rangle\right).$$

Then we can show $\langle A|a-1,b\rangle < \langle A|a,b\rangle$, as follows:

$$\begin{aligned}
\langle A|a,b\rangle &= K_\mathrm{d}^{-1}\left(\frac{K_\mathrm{d}+1+b-a}{a} + \frac{1}{a}\langle A|a-1,b\rangle\right)^{-1} \\
&> K_\mathrm{d}^{-1}\left(\frac{K_\mathrm{d}+1+b-a}{a} + \frac{1}{a}\left(\langle A|a-2,b\rangle+1\right)\right)^{-1} \qquad (\because \text{Eq. (S14)}) \\
&> K_\mathrm{d}^{-1}\left(\frac{K_\mathrm{d}+2+b-a+\langle A|a-2,b\rangle}{a-1}\right)^{-1} \\
&= K_\mathrm{d}^{-1}\left(\frac{K_\mathrm{d}+2+b-a}{a-1} + \frac{1}{a-1}\langle A|a-2,b\rangle\right)^{-1} \\
&= \langle A|a-1,b\rangle.
\end{aligned}$$

Manual for the computational package, ASSISTER

The computational package ASSISTER (Adaptive Simplification of StochastIc SystEm with Reversible binding; https://github.com/Mathbiomed/ASSISTER) contains three codes implemented in MATLAB: `LQSSA`, `QSSA_Threshold`, and `Gillespie_Reduction`. `LQSSA` calculates the L-state slQSSA for given A_T, B_T, K_d, and L. `QSSA_Threshold` is the function that determines which of the stQSSA and the slQSSA is valid, and calculates the number of needed states (L) for the slQSSA to ensure the smaller relative error than a tolerance for given K_d value (Fig 6). The function `Gillespie_Reduction` performs accurate stochastic simulations for any values of the parameters with the adaptive choice of the valid approximation method determined by using `QSSA_Threshold`. While `LQSSA` and `QSSA_Threshold` are auxiliary functions for the comprehensive code, `Gillespie_Reduction`, they can be used independently by users.

To perform `Gillespie_Reduction`, users need to enter the inputs for the Gillspie algorithm (the propensity function and stoichiometric vector for each reaction, the initial condition, and the time points) and the additional two inputs (the indices specifying rapid reversible binding reactions and the error tolerance). Then `Gillespie_Reduction` automatically constructs the reduced model with adaptive choice of the approximation strategy (i.e., the non-elementary propensities) within the given error tolerance.

The file `test` in the repository contains the codes for a running example (Fig 2). The function handle `prop_functions` represents the propensity functions of the reactions in the stochastic model. It can be adjusted by changing the function '`propensity_functions`'. All kinetic parameters should be entered together in this function as follows:

```
function lambda = propensity_functions(x)
   %fast reaction constants
   k_f = 100; % binding
   k_b = 1; % unbinding
   % slow reaction constants
   k_R = 0.1; %M_R transcription
   k_A = 0.001; %M_A transcription
   k_Rd = 0.001; %M_R Degradation
   k_Ad = 0.001; %M_A Degradation
   lambda(1) = k_f * x(1) * x(2); % D + P -> D:P
   lambda(2) = k_b * x(3); % D + P <- D:P
   lambda(3) = k_R * x(1); % D -> D + M_R
   lambda(4) = k_A * x(3); % D:P -> D:P + M_A
   lambda(5) = k_Rd * x(4); % M_R -> 0
   lambda(6) = k_Ad * x(5); % M_A -> 0
end
```

The variable `Stoi` is the $d \times R$ stoichiometric matrix where d is the number of species and R is the number of reactions. `x_init` is the initial condition for the species, and `tspan` is the vector of increasing time points at which simulated trajectories are measured. `rev_idx` is the $n \times 2$ matrix where n is the number of rapid reversible bindings to be reduced, and each column contains two indices that represent the pair of rapid reversible binding reactions. `err_tol` is the error tolerance of the approximation for the stochastic QSSA.

prop_functions = @propensity_functions;

```
Stoi = [-1 -1 1 0 0; 1 1 -1 0 0; 0 0 0 1 0; ...
        0 0 0 0 1; 0 0 0 -1 0; 0 0 0 0 -1]';
x_init = [10,10,0,0,0]';
tspan = 0:200:1600;
rev_idx = [1 2];
err_tol = 0.1;
```

Finally, the Gillespie algorithm with the reduced model can be performed by executing the following line:

```
Output = Gillespie_Reduction(Stoi, prop_functions, x_init, tspan,
rev_idx, err_tol)
```

`Output` contains the simulated trajectories of the species measured at each time point in `tspan` from the reduced model. Since the first (D) and second (P) species represent the binding molecules for the rapid reversible binding, they are not simulated in the reduced model. Instead, the trajectories of their corresponding total variables ($D + D{:}P$ and $P + D{:}P$) are stored in the corresponding rows (i.e., the first and second rows in this example) of `Output`. The zero vector is stored in the row corresponding to the bound complex (i.e., the third row in this example) for simplicity.

```
>> disp(Output) % print the entry in the variable 'Output'
    10   10   10   10   10   10   10   10   10   % trajectory of D+D:P
    10   10   10   10   10   10   10   10   10   % trajectory of P+D:P
     0    0    0    0    0    0    0    0    0   % dummy zero vector
     0    3    5    5    5    4    4    5    7   % trajectory of M_R
     0    1    2    4    5    6    9   10    8   % trajectory of M_A
```

Supplementary Tables

Table A. Propensity functions of the full model for the simple gene regulatory network (Fig 2a left)

Reactions	Propensity functions	Rate constants (s^{-1})
D + P → D:P	$\frac{k_\mathrm{f}}{\Omega}DP$	$k_\mathrm{f}/\Omega = 10^2, 10^2, 1, 10^2$ *
D:P → D + P	$k_\mathrm{b}D{:}P$	$1, 1, 10, 1$ *
D → D + $\mathrm{M_R}$	$k_\mathrm{R}D$	10^{-1}
D:P → D:P + $\mathrm{M_A}$	$k_\mathrm{A}D{:}P$	10^{-3}
$\mathrm{M_R} \to \emptyset$	$\tilde{k}_\mathrm{R}M_\mathrm{R}$	10^{-3}
$\mathrm{M_A} \to \emptyset$	$\tilde{k}_\mathrm{A}M_\mathrm{A}$	10^{-3}

* These four values are used for Figs 2b, 2c, 2d, and 5c, respectively.

[D, P, $D{:}P$, M_R, M_A] is initially [10, 10, 0, 0, 0], [15, 10, 0, 0, 0], [10, 10, 0, 0, 0], and [10, 10, 0, 0, 0] in Figs 2b, 2c, 2d, and 5c, respectively.

Table B. Propensity functions of the reduced models for the simple gene regulatory network obtained using the stQSSA (Fig 2a right) and the slQSSA (Fig 5c)

Reactions	Propensity functions		Rate constants (s^{-1})
	stQSSA	slQSSA	
$\emptyset \to \mathrm{M_R}$	$k_\mathrm{R}D_\mathrm{tq}$ [1]	$k_\mathrm{R}D_\mathrm{lq}$ [3]	10^{-1}
$\emptyset \to \mathrm{M_A}$	$k_\mathrm{A}D{:}P_\mathrm{tq}$ [2]	$k_\mathrm{A}D{:}P_\mathrm{lq}$ [4]	10^{-3}
$\mathrm{M_R} \to \emptyset$	$\tilde{k}_\mathrm{R}M_\mathrm{R}$		10^{-3}
$\mathrm{M_A} \to \emptyset$	$\tilde{k}_\mathrm{A}M_\mathrm{A}$		10^{-3}

[1] $D_\mathrm{tq} = \{(D_\mathrm{T} - P_\mathrm{T} - K_\mathrm{d}) + \sqrt{(D_\mathrm{T} - P_\mathrm{T} - K_\mathrm{d})^2 + 4D_\mathrm{T}K_\mathrm{d}}\}/2$

[2] $D{:}P_{tq} = \{(D_\mathrm{T} + P_\mathrm{T} + K_\mathrm{d}) - \sqrt{(D_\mathrm{T} - P_\mathrm{T} - K_\mathrm{d})^2 + 4D_\mathrm{T}K_\mathrm{d}}\}/2$

[3] $$D_\mathrm{lq} = \begin{cases} (D_\mathrm{T} - P_\mathrm{T} + 1)(D_\mathrm{T} - P_\mathrm{T} + P_\mathrm{T}K_\mathrm{d})(D_\mathrm{T} - P_\mathrm{T} + D_\mathrm{T}K_\mathrm{d} + 1)^{-1} & \text{if } D_\mathrm{T} \geq P_\mathrm{T} \\ (D_\mathrm{T}K_\mathrm{d})(P_\mathrm{T} - D_\mathrm{T} + D_\mathrm{T}K_\mathrm{d} + 1)^{-1} & \text{if } D_\mathrm{T} < P_\mathrm{T} \end{cases}$$

[4] $$D{:}P_\mathrm{lq} = \begin{cases} D_\mathrm{T} - (D_\mathrm{T} - P_\mathrm{T} + 1)(D_\mathrm{T} - P_\mathrm{T} + P_\mathrm{T}K_\mathrm{d})(D_\mathrm{T} - P_\mathrm{T} + D_\mathrm{T}K_\mathrm{d} + 1)^{-1} & \text{if } D_\mathrm{T} \geq P_\mathrm{T} \\ D_\mathrm{T} - (D_\mathrm{T}K_\mathrm{d})(P_\mathrm{T} - D_\mathrm{T} + D_\mathrm{T}K_\mathrm{d} + 1)^{-1} & \text{if } D_\mathrm{T} < P_\mathrm{T} \end{cases}$$

[M_R, M_A] is initally [0, 0] and $P_\mathrm{T} = 10$. $D_\mathrm{T} = 10, 15, 10, 10$ and $K_\mathrm{d} = 10^{-2}, 10^{-2}, 10, 10^{-2}$ in Figs 2b, 2c, 2d, and 5c, respectively.

Table C. Propensity functions of the full model for the biological oscillator (Fig 3a top)

Reactions	Propensity functions	Rate constants (s^{-1})
A + R → A:R	$\frac{k_\mathrm{f}}{\Omega}AR$	$k_\mathrm{f}/\Omega = 10^6$
A:R → A + R	$k_\mathrm{b}A{:}R$	10^2
A → A + M	$\alpha_\mathrm{M}A$	20
M → M + P_1	$\alpha_\mathrm{P}M$	1.5
P_1 → P_1 + P_2	$\alpha_\mathrm{P}P_1$	1
P_2 → P_2 + P_3	$\alpha_\mathrm{P}P_2$	1
P_3 → P_3 + R	$\alpha_\mathrm{R}P_3$	1
M → ∅	$\beta_\mathrm{M}M$	1
P_i, $i = 1, 2, 3 \to \emptyset$	$\beta_\mathrm{P}P_i$	1
R → ∅	$\beta_\mathrm{R}R$	$0.02, 1, 0.02^*$
A:R → A	$\beta_\mathrm{R}A{:}R$	$0.02, 1, 0.02^*$

* These three values are used for Figs 3b, 3d, and 5d, respectively.
$[A,\ R,\ A{:}R,\ M,\ P_1,\ P_2,\ P_3]$ is initially [5, 5, 0, 0, 0, 0, 0].

Table D. Propensity functions of the reduced models for the biological oscillator obtained using the stQSSA (Fig 3a bottom) and the slQSSA (Fig 5d)

Reactions	Propensity functions		Rate constants (s^{-1})
	stQSSA	slQSSA	
∅ → M	$\alpha_\mathrm{M}A_\mathrm{tq}{}^1$	$\alpha_\mathrm{M}A_\mathrm{lq}{}^2$	20
M → M + P_1	$\alpha_\mathrm{P}M$		1.5
P_1 → P_1 + P_2	$\alpha_\mathrm{P}P_1$		1
P_2 → P_2 + P_3	$\alpha_\mathrm{P}P_2$		1
P_3 → P_3 + R_T	$\alpha_\mathrm{R}P_3$		1
M → ∅	$\beta_\mathrm{M}M$		1
P → ∅	$\beta_\mathrm{P}P$		$0.02, 1, 0.02^*$
R_T → ∅	$\beta_\mathrm{R}R_\mathrm{T}$		$0.02, 1, 0.02^*$

[1] $A_\mathrm{tq} = \{(A_\mathrm{T} - R_\mathrm{T} - K_\mathrm{d}) + \sqrt{(A_\mathrm{T} - R_\mathrm{T} - K_\mathrm{d})^2 + 4A_\mathrm{T}K_\mathrm{d}}\}/2$

[2] $A_\mathrm{lq} = \begin{cases} (A_\mathrm{T} - R_\mathrm{T} + 1)(A_\mathrm{T} - R_\mathrm{T} + R_\mathrm{T}K_\mathrm{d})(A_\mathrm{T} - R_\mathrm{T} + A_\mathrm{T}K_\mathrm{d} + 1)^{-1} & \text{if } A_\mathrm{T} \geq R_\mathrm{T} \\ (A_\mathrm{T}K_\mathrm{d})(R_\mathrm{T} - A_\mathrm{T} + A_\mathrm{T}K_\mathrm{d} + 1)^{-1} & \text{if } A_\mathrm{T} < R_\mathrm{T} \end{cases}$

* These three values are used for Figs 3b, 3d, and 5d respectively.
$[R_\mathrm{T},\ M,\ P_1,\ P_2,\ P_3]$ is initially [5, 0, 0, 0, 0], $A_\mathrm{T} = 5$, and $K_\mathrm{d} = 10^{-4}$.

Table E. Propensity functions of the full model for the bistable switch (Fig 4a top) [7, 8]

Reaction	Propensity function	Rate constants (s^{-1})
M + B → M:B	$\frac{k_{\mathrm{f}}}{\Omega} MB$	$k_{\mathrm{f}}/\Omega = 10^4, 10, 10^{4*}$
M:B → M + B	$k_{\mathrm{b}} M{:}B$	$10^1, 10^2, 10^{1*}$
M → P	$k_{\mathrm{p}} M$	0.6
M:B → M + P	$k_{\mathrm{p}} M{:}B$	0.6
P → M	$\left(k_1 + (k_2 - k_1)\frac{M}{K_{\mathrm{D}}+M}\right) DP$	$k_1 = 0.05$, $k_2 = 1.1$

* These three values are used for Figs 4b, 4d, and 5e, respectively.
$[M,\ B,\ M{:}B,\ P]$ is initially [0, 10, 0, 30], $D = 1$, $K_{\mathrm{D}} = 0.1$.

Table F. Propensity functions of the reduced models for the bistable switch obtained using the stQSSA (Fig 4a bottom) and the slQSSA (Fig 5e) [7, 8]

Reactions	Propensity functions		Rate constants (s^{-1})
	stQSSA	slQSSA	
$\mathrm{M_T}$ → P	$k_{\mathrm{p}} M_{\mathrm{T}}$		0.6
P → $\mathrm{M_T}$	$\left(k_1 + (k_2 - k_1)\frac{M_{\mathrm{tq}}}{K_{\mathrm{D}}+M_{\mathrm{tq}}}\right) DP^1$	$\left(k_1 + (k_2 - k_1)\frac{M_{\mathrm{lq}}}{K_{\mathrm{D}}+M_{\mathrm{lq}}}\right) DP^2$	$k_1 = 0.05$, $k_2 = 1.1$

[1] $M_{\mathrm{tq}} = \{(M_{\mathrm{T}} - B_{\mathrm{T}} - K_{\mathrm{d}}) + \sqrt{(M_{\mathrm{T}} - B_{\mathrm{T}} - K_{\mathrm{d}})^2 + 4M_{\mathrm{T}}K_{\mathrm{d}}}\}/2$

[2] $$M_{\mathrm{lq}} = \begin{cases} (M_{\mathrm{T}} - B_{\mathrm{T}} + 1)(M_{\mathrm{T}} - B_{\mathrm{T}} + B_{\mathrm{T}}K_{\mathrm{d}})(M_{\mathrm{T}} - B_{\mathrm{T}} + M_{\mathrm{T}}K_{\mathrm{d}} + 1)^{-1} & \text{if } M_{\mathrm{T}} \geq B_{\mathrm{T}} \\ (M_{\mathrm{T}}K_{\mathrm{d}})(B_{\mathrm{T}} - M_{\mathrm{T}} + M_{\mathrm{T}}K_{\mathrm{d}} + 1)^{-1} & \text{if } M_{\mathrm{T}} < B_{\mathrm{T}} \end{cases}$$

$[M_{\mathrm{T}},\ P]$ is initially [0, 30], $D = 1$, $K_{\mathrm{D}} = 0.1$, and $B_{\mathrm{T}} = 10$. $K_{\mathrm{d}} = 10^{-3}, 10, 10^{-3}$ in Figs 4b, 4d, and 5e, respectively.

Supplementary Figures

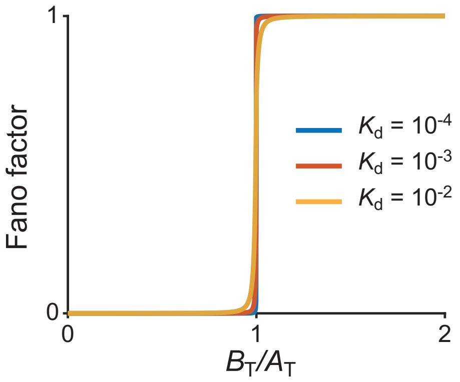

Fig A. The Fano factor of A (F_A) is between 0 and 1. As K_d decreases, F_A becomes closer to the step function, which is 0 when $A_T > B_T$ and 1 when $A_T \leq B_T$. Since the relative error of the stQSSA for A to the stochastic QSSA (R_A) satisfies $F_A S_A \leq R_A \leq 2F_A S_A$ (Eq. (7) in the main text), R_A is almost 0 when $B_T < A_T$ while R_A is between S_A and $2S_A$ when $B_T \geq A_T$. Therefore, when A tightly binds with B and $B_T \geq A_T$, R_A mainly depends on S_A (Fig 1d and 1e). Here, $A_T = 100$. See Supplementary Methods for the proof that F_A is always between 0 and 1.

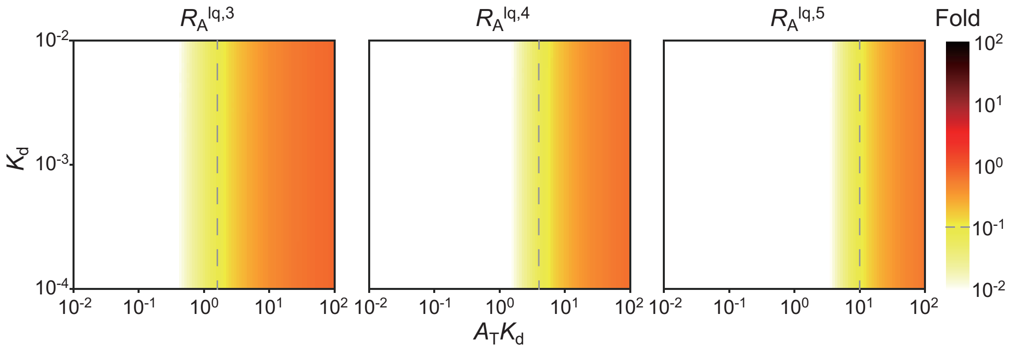

Fig B. slQSSAs with more states approximate the stochastic QSSA more accurately. The heat maps of the relative errors ($R_A^{lq,k} = \left|\frac{A_{lq}^k - \langle A\rangle}{\langle A\rangle}\right|$) when the k-state slQSSA (A_{lq}^k) approximates the stochastic QSSA for A ($\langle A\rangle$) for $k = 3, 4, 5$. See Eq. (19) in the main text for a detailed definition of A_{lq}^k. Color in the heat maps represents the maximum value of $R_A^{lq,k}$ for each $A_T K_d$ and K_d when B_T varies. As more states are used (i.e., k increases), A_{lq}^k accurately approximates $\langle A\rangle$ in the wider regions. Specifically, when $A_T K_d$ is less than 2, 5, and 10 (i.e., left side of the dashed lines in the heat maps), $R_A^{lq,3}$, $R_A^{lq,4}$ and $R_A^{lq,5}$ are less than 0.1, respectively. This indicates that one can accurately approximate $\langle A\rangle$ with relative error of less than 0.1 regardless of the values of A_T, B_T, and K_d by adaptively using both A_{tq} and A_{lq}^5 because the relative error of A_{tq} is less than 0.1 when $A_T K_d > 10$.

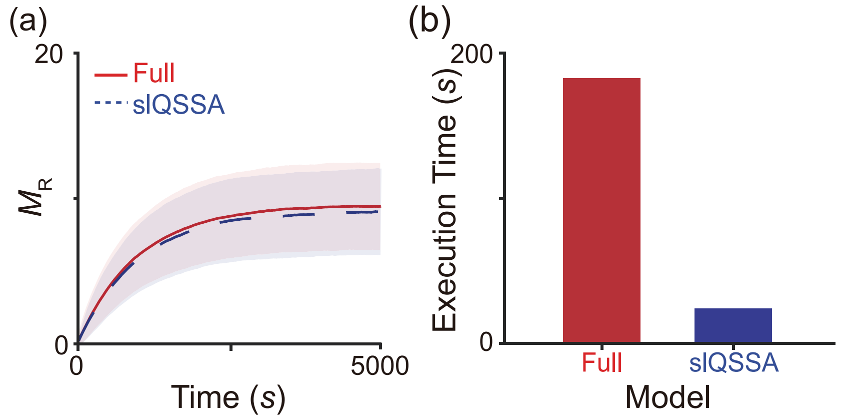

Fig C. Simulation benchmark comparing the full model and the reduced model using the slQSSA. (a) Simulated trajectories of M_{R} with the full model and the reduced model for the simple gene regulatory network (Figs 2a and 5c), using GillesPy2, one of the major, standard software suites for Gillespie type simulations [9]. The lines with colored ranges represent the mean $\pm$ standard deviation of 10^4 trajectories. The results are consistent with Fig 5c generated using MATLAB. All parameters are the same as in Fig 5c. **(b)** Execution times for the 10^4 repetitive simulations with the full model (182.5s) and the slQSSA model (24.0s). The simulation with the reduced model was approximately 7.5 times faster than the full model. The simulations were conducted with Intel(R) Core(TM) i9-10900K CPU @ 3.70GHz.

References

1. Rao CV, Arkin AP. Stochastic chemical kinetics and the quasi-steady-state assumption: Application to the Gillespie algorithm. J Chem Phys. 2003;118(11):4999–5010.

2. Cao Y, Gillespie DT, Petzold LR. The slow-scale stochastic simulation algorithm. J Chem Phys. 2005;122(1):014116.

3. Cao Y, Gillespie DT, Petzold LR. Accelerated stochastic simulation of the stiff enzyme-substrate reaction. J Chem Phys. 2005;123(14):144917.

4. Gómez-Uribe CA, Verghese GC, Tzafriri AR. Enhanced identification and exploitation of time scales for model reduction in stochastic chemical kinetics. J Chem Phys. 2008;129(24):244112.

5. Sontag ED, Zeilberger D. A symbolic computation approach to a problem involving multivariate Poisson distributions. Adv Appl Math. 2010;44(4):359–377.

6. Anderson DF, Craciun G, Kurtz TG. Product-form stationary distributions for deficiency zero chemical reaction networks. Bull Math Biol. 2010;72(8):1947–1970.

7. Thron CD. A model for a bistable biochemical trigger of mitosis. Biophys Chem. 1996;57(2-3):239–251.

8. Thron CD. Bistable biochemical switching and the control of the events of the cell cycle. Oncogene. 1997;15(3):317–325.

9. Drawert B, Hellander A, Bales B, Banerjee D, Bellesia G, Daigle BJ Jr, et al. Stochastic simulation service: bridging the gap between the computational expert and the biologist. PLoS Comput Biol. 2016;12(12):e1005220.
